# Supplementary material for: Image-Guided Liver Stereotactic Body Radiotherapy Using VMAT and Real-Time Adaptive Tumor Gating: Evaluation of the Efficacy and Toxicity for Hepatocellular Carcinoma
Source: Cancers (Basel). 2021 Sep 28;13(19):4853. doi: 10.3390/cancers13194853 (PMC8507769; doi:10.3390/cancers13194853)
Supplement: Supplementary file 1 [file cancers-13-04853-s001.zip › cancers-1369045-supplementary.pdf]

Table S1. Prospective studies [34,36–47,35,48–50] evaluating SBRT for HCC: LC, local control; OS, overall survival; PFS, progression free survival; gastro intestinal toxicities = gastric ulcer, duodenal ulcer ; liver toxicities = hepatic pains, ascites, decompensation of cirrhosis, disturbed liver function, RILD and non classic RILD.

Studies	Number of Patients	Median Size of Lesion (mm)	Average Volume Treated (cm3) GTV/PTV	Total Dose (Gy)	Nb of Fraction	Median Follow up (Months)	Local Control 1 yr/2 yrs (%)	OS 1 yr/2 yrs (%)	PFS 1 yr/2 yrs (%)	Gastrointestinal Toxicities (Grade > 2)	Liver Toxicity (Grade > 2)
**Tse et al., 2007 Canada** [18]	31	-	173/-	24–54	6	17.6	65/-	48/-	-	G5 = 3% G4 = 6.5%	G3 = 25.8%
Mendez-Romero et al., 2009 The Netherlands [19]	8	32	22/-	25–37.5	3–5	12.9	94 /82	-	-	-	G3 = 37.5% G5 = 12.5%
**Cardenes et al., 2010, Mexico** [20]	17	40	34/-	36–48	3	24	100/100	75/60	-	-	RILD = 17.6%
Kang et al., 2012 Korea [21]	47	29	-	42–60	3	17	-/94.6	-/68.7	-/33.8	G4 = 4,3%	G3 bio = 15% G3 clinical = 4%
Bujold et al., 2013 Canada [22]	102	72	117/283.5	24–54	6	31	87.2/-	55/-	-	G3 = 2% G5 = 1%	G3 = 1% G4 = 1% G5 = 6%
Scorsetti el al, 2015 Italy [23]	43	48	-/169.7	36–75	3–6	8	85.8/64.4	77.9/45.3	41/-	-	G3 = 16%
Kim et al., 2016 Korea [24]	18	20.5	-/79.9	36–60	4	23	77.8/71.3	94.4/69.3	55.6/49.4	-	-

Table S2. Retrospective studies evaluating SBRT for HCC: OS = overall survival, PFS = progression free survival, gastro intestinal toxicities = gastric ulcer, duodenal ulcer; liver toxicities = hepatic pains, ascites, decompensation of cirrhosis, disturbed liver function, RILD and non classic RILD.

Retrospective Studies	Number of Patients	Median Lesion Size (mm)	Average Volume Treated (cm³) GTV/PTV	Total Dose (Gy)	Number of Fractions	Median Follow-up (Months)	LC 1 Year/2 Years (%)	OS 1 Year/2 Years (%)	PFS 1 Year/2 Years (%)	Gastrointestinal Toxicity (Grade > 2)	Liver Toxicity (Grade > 2)
Blomgren et al. 1995-Sweden	8	-	110/-	30	2–3	12	100–95%	-	-	-	G5 = 37.5%
Choi et al. 2008-Korea	31	-	25.2/-	30–39	3	10.5	71.9/-	81.4/-	-	-	RILD = 6.5%
Seo et al. 2010-Korea	38	-	40.5/-	33–57	3–4	15	78.5/66.4	68.4/61.4	46.4/37.5	-	-
Kwon et al. 2010-Korea	42	-	77/-	30–39	3	28.7	59.6/-	92.9/-	72/-	-	G4 = 1% RILD = 2%
Andolino et al. 2011-USA	60	31	-	24–48	3–5	27	-/90	-/67	-/48	-	RILD = 6.7% G5 = 3.3%
Dewas et al. 2012-France	42	40	57.5/138.2	27–45	3	15	89/89	72/42.4	56/38	G3 = 12%	RILD = 7.1%
Bibault et al. 2013-France	75	37	130.7/-	45	3	10	89.8/89.8	78.5/50.4	-	G4 = 1.3%	NC RILD = 6.6%
Yoon et al.	93	20	8.6/40.8	30–60	3–4	25.7	94.8/92.1	86/53.8	87.9/72.2	-	G5 = 1%
2013-Korea											
Jang et al. 2013-Korea	82	30	-	33–60	3	30	-/87	-/63	-	G3–4 = 6%	NC RILD = 7%
Sanuki et al. 2014-Japan	48<br>132	27<br>24	8.9/40.4<br>7.2/37.1	35<br>40	5<br>5	31<br>23	97.5/90.7<br>100/94.2	95/83<br>95.7/82.5	97/97<br>95.9/90.1	-	G5 = 1% RILD = 9.4%
Lo et al. 2014-Taiwan	53	43	-	40	4–5	13	73.3/66.8	70.1/45.4	-	G 3–4 = 5.5%	RILD = 1.8%
Takeda et al. 2014-Japan	63	26	-	35–40	5	31	100/95	100/87	-	-	16%
Huertas et al. 2015-France	77	24	11.7/-	45	3	12	99/99	81.8/56.6	69.3/44.4	G3 = 1.3% G4 = 1.3% G5 = 1.3%	G3 = 3.9%
Baumann et al. 2018-USA	37	27	-	50	5	14	95/-	87/-	66/-	G3 = 2.7%	G3 = 8%
Zhang et al. 2018-China	28	21	-	35–60	3–6	36	96.4–92.9	92.9/85.7	-	-	G4bio = 3.5%
Yeung et al. 2018-China	31	33	-	45	3–5	18.3	84/-	84/-	49	Acute = 3.2% Delayed = 0%	Acute = 22.6% Delayed = 6.4% NC RILD = 19%
Scher et al. 2019-France	136	22	-/41.3	45	3	13	94.5–91%	79.8–63.5%	61.3–39.4%	Acute = 3% Delayed = 2%	RILD = 14%
